# Novel minimally invasive treatments for lower urinary tract symptoms: a systematic review and network meta-analysis

**DOI:** 10.1590/S1677-5538.IBJU.2023.0016

**Published:** 2023-05-20

**Authors:** Robertus Arnoldus Antonius van Kollenburg, Luigi Antonio Maria Johannes Gerardus van Riel, Daniel Martijn de Bruin, Theodorus Maria de Reijke, Jorg Reinier Oddens

**Affiliations:** 1 University of Amsterdam, Biomedical Engineering and Physics Amsterdam UMC Department of Urology Netherlands Department of Urology, Amsterdam UMC, University of Amsterdam, Biomedical Engineering and Physics, Netherlands

**Keywords:** Systematic Review [Publication Type], Minimally Invasive Surgical Procedures, Prostate

## Abstract

**Purpose:**

To review and compare the effectivity of novel minimally invasive treatments (MITs) to transurethral resection of the prostate (TURP) for the treatment of lower urinary tract symptoms (LUTS) in men.

**Methods:**

Medline, Embase, and Cochrane databases were searched from January 2010 to December 2022 for randomized controlled trials (RCTs) evaluating MITs, compared to TURP or sham, in men with LUTS. Studies were assessed by risk of bias tool, and evidence by GRADE. Functional outcomes by means of uroflowmetry and IPSS were the primary outcomes, safety and sexual function were secondary outcomes. As part of this review, a network meta-analysis (NMA) was conducted. MITs were ranked based on functional outcome improvement probability.

**Results:**

In total, 10 RCTs were included, evaluating aquablation, prostatic urethral lift, prostatic artery embolization (PAE), convective water vapor thermal treatment or temporary implantable nitinol device. All MITs showed a better safety profile compared to TURP. Functional outcome improvement following aquablation were comparable to TURP. In the NMA, aquablation was ranked highest, PAE followed with the second highest probability to improve functional outcomes. Other novel MITs resulted in worse functional outcomes compared to TURP. Level of evidence was low to very low.

**Conclusions:**

Five MITs for treatment of LUTS were identified. Aquablation is likely to result in functional outcomes most comparable to TURP. Second in ranking was PAE, a technique that does not require general or spinal anesthesia. MITs have a better safety profile compared to TURP. However, due to high study heterogeneity, results should be interpreted with caution.

## INTRODUCTION

Bladder outlet obstruction is associated with lower urinary tract symptoms (LUTS) in men. If medical treatment fails to provide relief, a surgical procedure may be considered (1). Transurethral resection of the prostate (TURP) has proven its success in LUTS improvement and is considered the standard of care (1). However, it requires general or spinal anesthesia and hospital admission. Furthermore, it comes with side effects, such as retrograde ejaculation, and the risk of complications such as hematuria, clot retention, and urethral stricture (1).

Less invasive treatments such as transurethral microwave treatment, transurethral needle ablation, and interstitial laser coagulation treatments showed that minimally invasive procedures provided an improved safety profile, but functional outcomes were inferior to TURP (1-3). Therefore, they are not recommended in the 2021 EAU guidelines (1).

Recently, several novel minimal invasive treatments (MITs) have been developed using new approaches or energy sources for the treatment of LUTS. These MITs include the use of steam, waterjet, anchors, prostatic vascular embolization and temporarily implanted devices (4-8). Several studies have been performed to study the safety and functional outcomes of MITs, including randomized controlled trials (RCTs) (9-12). In these studies, MITs have been compared to either TURP or sham. However, there is a lack of direct or indirect comparisons between different MITs, challenging the determination of a preferred treatment.

This review aims to provide an overview of trials comparing MITs with TURP or sham. Using a network meta-analysis (NMA) approach, the comparative effectivity of these techniques and standard of care were analyzed.

## METHODS AND METHODS

This systematic review is performed in accordance with the PRISMA guidelines (13). The review was registered at Prospero (CRD42020208039).

### Eligibility criteria

A search was performed to identify randomized controlled trials that studies novel minimally invasive treatments as intervention and TURP or sham as control for men with lower urinary tract symptoms. The search was limited from January 2010 to the present to identify recent MITs. Earlier developed techniques are no longer recommended in the guidelines. Non-English articles were considered. The search was limited using the exclusion of female in the title or abstract, to improve the quality of the search.

### Information sources

A systematic search was performed on MEDLINE (PubMed), Embase (Ovid), and Cochrane (Cochrane). Included records were screened for secondary interesting studies.

### Search strategy

A systematic search was performed on December 7th 2022 using the following search: Randomized controlled trial OR randomi* OR trial (all title, abstract, keyword) AND Lower urinary tract symptom* OR benign prostat* (all title, abstract, keyword) AND Minimally invasive surgery OR minimally invasive procedure OR novel treatment OR Aquablation OR rezum OR urolift OR prostatic urethral lift OR emboli* OR surgical intervention OR laser OR new treatment (all terms) NOT female OR woman OR women (all title or abstract), limited to the period January 2010 to December 7th 2022.

### Data management and selection

Records were managed in Endnote (version 20). Duplicates were removed by R.K. using EndNote’s duplicate identification and removal tool. All identified records were independently reviewed by two reviewers (R.K. and L.R.). Any disagreements were settled by consensus and a third independent reader was consulted when necessary (J.O.).

### Data extraction

Data extraction was performed independently by two reviewers (R.K. and L.R.) according to the prior established protocol. In case of missing data, corresponding authors were contacted. Plot digitizer (version 2.6.9) was used to extract data from figures when outcomes were not explicitly mentioned in the full text of included articles (14).

### Risk of Bias and quality of evidence assessment

The risk of bias was evaluated using the Cochrane Risk of Bias 2 tool (15). Two reviewers (R.K. and L.R.) performed the risk of bias assessment independently. Disagreement was solved by consensus and a third independent reader was consulted when necessary (J.O.). The quality of evidence was evaluated using GRADE.

### Statistical Analysis

The baseline characteristics (e.g., age, prostate volume, Qmax, IPSS) and data of the included studies were descriptively summarized. The continuous outcomes (e.g., Qmax, IPSS) were summarized by the quantitative information provided by the included studies, including means plus standard deviations (SD). Dichotomous variables were examined in the descriptive analysis with proportions and event rates.

### Network meta-analysis (NMA)

For each outcome, an NMA with a Bayesian approach was conducted using a randomeffects model. In the absence of direct evidence for given comparisons, the indirect comparisons provided the estimates. In presence of both direct and indirect evidence, the NMA model provided a mixed-effect estimate (16). Since the NMA resulted in a star-shaped network, all the evidence of comparisons between the interventions was indirect, except for active treatments compared to the common comparator (i.e., TURP). Therefore, network consistency could not be assessed. We estimated the relative ranking of the different treatments using the distribution of the ranking probabilities and the surface under the cumulative ranking (SUCRA) curve (17). The larger the SUCRA for a specific treatment, the higher its ranking among the available treatment options. Direct and indirect meta-analysis was conducted in STATA, release 12 (StataCorp, College Station, Texas) using the network meta-analysis command (18).

## RESULTS

### Systematic search

**Figure 1:**
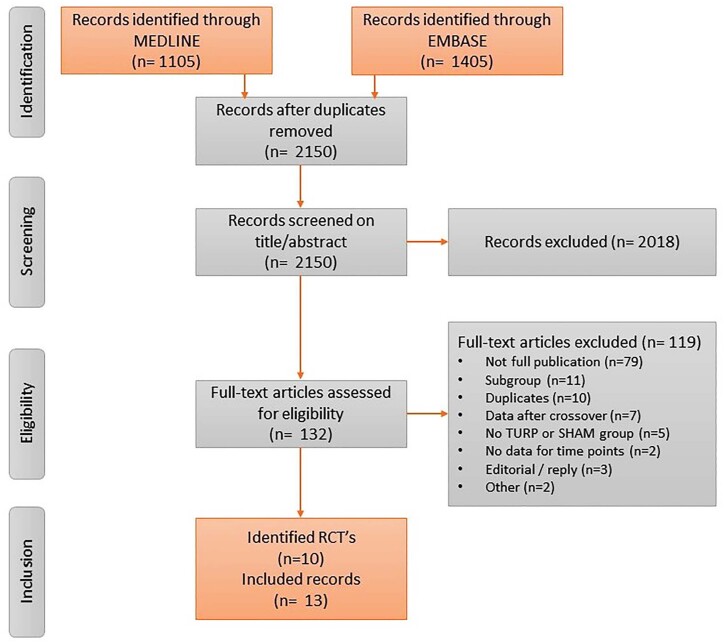
Screening overview, based on the PRISMA method.

**Figure 2:**
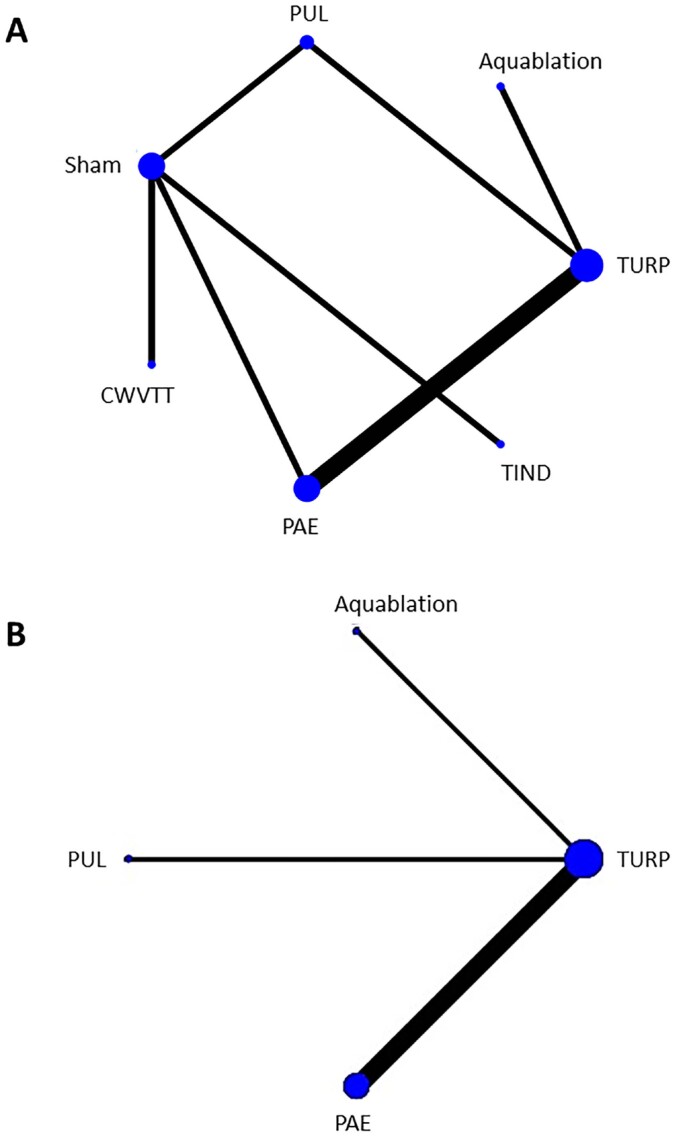
Network diagrams showing the network at A) three and B) 12 months. The dots indicate each included treatment. The lines that connect the dots indicate the direct comparisons between treatment groups. Thickness of the lines represents the available number of trials.

The literature search identified 2150 unique records. Based on title and abstract 2018 records were excluded. The remaining 132 records underwent full-text assessment for eligibility. Finally, 13 articles covering ten studies were included (9-11, 19-28) (Figure-1). Five MITs were identified: Aquablation, prostatic urethral lift (PUL), convective water vapor thermal therapy (CWVTT), prostatic artery embolization (PAE), and temporary implantable nitinol device (TIND). Six studies compared MIT with TURP, and four studies compared MIT with sham (Table-1, Figure-2). For the primary outcomes at three months, all identified studies were included for analysis. At twelve months, only six trials were included, since sham group cross-over was at three months (9, 20, 27, 28).

**Table 1:** Details and baseline patient characteristics of included studies.

Studies	Inclusion period	Region (n of centers)	Intervention (n)	Control (n)	Age (years±SD)	Prostate volume (mL±SD)	Qmax (mL/ s±SD)	PVR (mL±SD)	IPSS (±SD)	QoL (±SD)	IIEF-5 (±SD)
Aquablation, Gilling et al. (4)	2015-2016	International (17)	Aquablation (117)	TURP (67)	66.0±7.3	51.4±16.2	9.4±3.0	97±79	22.9±6.0	4.8±1.1	17.2±6.5
PUL, Roehrborn et al. (2)	2011	International (19)	PUL (140)	Sham (66)	67±8.6	44.5±12.4	8.9±2.2	85.5±69.2	22.2±5.4	4.6±1.1	13.0±8.4
PUL, Sønksen et al. (11)	2012-2013	International (10)	PUL (45)	TURP (35)	63±6.8	38±12	9.2±3.5	86±72	22±5.7	4.7±1.1	20±4.9
CWVTT, McVary et al. (23)	2013-2014	USA (15)	CWVTT (136)	Sham (61)	63.0±7.1	45.8±13.0	9.9±2.3	82.0±51.5	22.0±4.8	4.4±1.1	NR
PAE, Insausti et al. (26)	2014-2017	Spain (1)	PAE (23)	TURP (22)	72.4±6.2	60.0±4.5	7.7±0.54	82.2±41.9	26.6±0.3	4.5±0.2	15.7±7.2
PAE, Abt et al. (25)	2014-2017	Switzerland (1)	PAE (48)	TURP (51)	65.7±9.3	51.2±16.5	7.5±4.1	168.5±183	19.4±6.4	4.0±1.0	15.15
PAE, Gao et al. (21)	2007-2012	China (1)	PAE (57)	TURP (57)	67.7±8.7	64.7±19.7	7.8±2.5	126.9±68.8	22.8±5.9	4.8±0.8	NR
PAE, Pisco et al. (28)	2014-2018	Portugal (1)	PAE (40)	Sham (40)	63.9±5.83	83.1±47.7	7.5	139.2±105.4	25.9±3.9	4.4±0.5	NR
PAE, Carnevale et al. (24)	2010-2012	Brazil (1)	PAE (15)	TURP (15)	63.5±8.7	63.0±17.8	7.0±3.6	127±99.9	25.3±3.6	4.7±0.6	14.3±6.8
TIND, Chughtai et al. (27)	2015-2018	US (14) and Canada (2)	TIND (128)	Sham (57)	61.5±6.5	43.4±15.5	8.7±3.3	61.6±55.5	22.1±6.8	4.6±1.3	NR

CWVTT = Convective Water Vapor Thermal Therapy; IIEF = International Index of Erectile Function; IPSS = International Prostate Symptom Score; NR = Not Reported; PAE = Prostatic Artery Embolization; PVR = Post-Void Residual; PUL = Prostatic Urethral Lift; Qmax = Peak Urinary Flow; QoL = Quality of Life; SD = standard deviation

### Working mechanisms of the identified minimally invasive techniques.

Aquablation uses a high-velocity water jet to ablate prostate tissue. The technique has a transurethral approach and is automated using ultrasound image-guidance (4). Hemostasis following ablation is accomplished by bipolar coagulation.

PUL uses anchor shaped implants that are introduced through the lobe until the capsule. Under compression of the lobe an end-piece is placed on the monofilament, reducing the obstruction without tissue removal (6).

CWVTT is based on water vapor injection in the prostatic lobes. Water vapor is introduced in the lobe during multiple nine second treatments, using a specific cystoscope with puncture needle. Due to the convective properties of water vapor, the treatment is naturally limited to the prostate capsule (5).

PAE is based on artery embolization. A sheath is placed in the common femoral artery. The prostate artery supply is identified using angiography. When identified microspheres are introduced, various sizes are used in the included studies. The procedure is repeated on the contralateral side (7).

TIND is a device that is inserted into the urethra and expanded. By exerting pressure on the prostatic tissue, ischemic necrosis is induced, incising the prostate, and reshaping it. The device is generally removed after over the period of five to seven days (8).

### Characteristics of the identified studies

-1 lists the included studies and their main characteristics. A total of 117, 185, 136, 183, 128, and 247 patients were included for treatment with Aquablation, PUL, CWVTT, PAE, TIND, or TURP, respectively. There were no substantial differences in baseline characteristics of included patients (Table-1). The only notable difference was seen in study by Abt et al. as IPSS and QoL were slightly better compared to other studies (25). However, Qmax and PVR of the same patients were worse than most other studies, which is contradictive. Inclusion and exclusion criteria did not substantially differ between studies, except for a median lobe as exclusion criteria for PUL (see supplementary Table-1).

### Risk of Bias

The risk of bias for the included studies was evaluated separately for objective outcomes (Qmax and post-void residual), subjective outcomes (IPSS and QoL) and adverse events (see supplementary Table-2). Among the included studies, two studies scored “low risk” on all domains and outcomes (9,10). While two studies scored “high risk” on the objective and subjective outcomes (26, 27). In the study by Insausti et al. prostates up to 120cc were included. However, data of patients with a prostate of >100cc were excluded during analyses, as TURP is only indicated for prostates ≤ 100cc according to guidelines. This exclusion might have introduced reporting bias, as outcomes in the intervention group might have been worse. In the study by Chughtai et al. a 29-30% loss of follow-up was seen in both arms during the first three months, which might have impacted outcomes, as the reason behind the lost to follow-up is unknown. Therefore, the study may have resulted in less reliable outcomes. The remaining studies had some concerns in one or multiple domains and/or outcomes.

### Peri-operative outcomes

Aquablation is performed under general or spinal anesthesia (Table-2) (4). PUL, CWVTT, PAE and TIND are performed under local anesthesia of the prostate or puncture site (11, 20, 21, 24-29). Procedure times were 32.8±16.5 minutes for Aquablation, 55±17 to 66.2±23.8 for PUL, and 75 (60-90) to 144±50.1 minutes for PAE. Procedural time for TIND was not reported. All Aquablation procedures required hospital admission, with an average stay of 1.4 days, while about half of the PAE procedures required hospital admission for 2.2 days. PUL, CWVTT, TIND, and about half of the PAE treatments were performed in an outpatient setting or day-care admission.

**Table 2:** Peri-operative characteristics.

Studies	Anesthesia	Setting	Procedural time (min), mean±SD	Hospital stays (days), mean±SD	Spontaneous voiding or bladder catheter (days), mean±SD)	Median lobe treatment possible
Aquablation, Gilling et al. (4)	General (94%) and spinal (6%)	Operating room	32.8±16.5	1.4±0.7	Bladder catheter, median of 1 day	Yes
PUL, Roehrborn et al. (2)	168/169 local using diazepam and Lidocaine gel in 164 and prostatic block in 4, general anesthesia in 37#	Outpatient	66.2±23.8	NR	68% spontaneous void, 32% bladder catheter with a mean duration 0.9 days	No
PUL, Sønksen et al. (11)	General (86%), spinal (13%), topical (1%)	Operating room	55±17	1.0±0.9	45% bladder catheter > 24h	No
CWVTT, McVary et al. (23)	Oral sedation (68.9%), prostate block (20.9%), conscious intravenous (10.2%)	Outpatient	NR	NR	90.4% bladder catheter 3.4±3.2 days	Yes
PAE, Insausti et al. (26)	Local (skin)	NR	138.7±51.9	1±0	No, if spontaneous void pre-PAE	Yes
PAE, Abt et al. (25)	Local (skin)	Clinical	122.2±25.8	2.2±0.6	Bladder catheter, 1.3±1.4	Yes
PAE, Gao et al. (21)	Local (skin)	NR	89.7±17.1	2.9±1.6	35.2% bladder catheter	NR
PAE, Pisco et al. (28)	Local (skin)	NR	75 (60-90)*	NR	NR	NR
PAE, Carnevale et al. (24)	Local (skin)	Outpatient	144.8±50.1	0±0.25	NR	NR
TIND, Chughtai et al. (27)	Local (27%), IV sedation (66%), or general anesthesia (7%)	Outpatient/ clinical	NR	Same day discharge	No bladder catheter	Exclusion criteria

# Australian cohort received general anesthesia as standard of care

* Median time,

NR = not reported

CWVTT = Convective Water Vapor Thermal Therapy; NR = Not Reported; PAE = Prostatic Artery Embolization; PUL = Prostatic Urethral Lift; SD = standard deviation;TURP = Transurethral Resection of the Prostate

### Functional outcomes

#### Qmax

**Figure 3:**
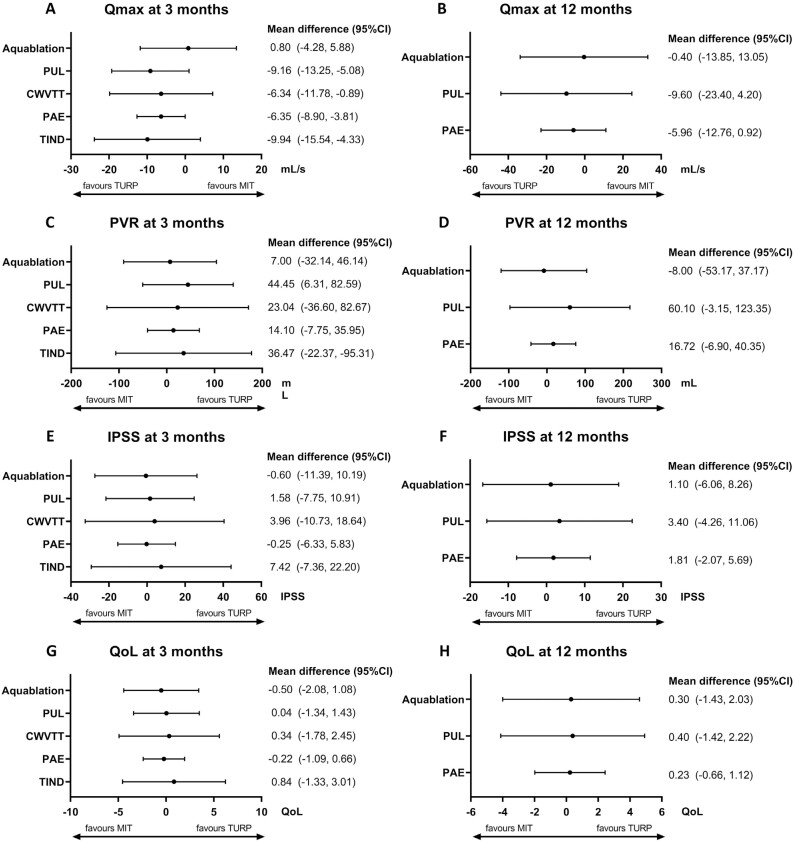
Network meta-analysis outcomes comparing MIT with TURP for Qmax, PVR, IPSS and QoL at three and twelve months. On the right side of each graphs the mean difference and 95% confidence interval of the specific outcome are shown. The arrow under each graph shows per technique whether the MIT or TURP is favored.

An indirect comparison of MITs with TURP showed that Aquablation provided the greatest Qmax improvement compared to the other MITs, with Qmax improvement following Aquablation being comparable to TURP at both 3- and 12-months follow-up, with a mean difference (MD) of 0.80; (95%CI: -4.25,5.88) and an MD of -0.40 (95% CI: -13.85, 13.05) (Figures 3A and B). At 3 months, PAE, CWVTT, and PUL resulted in significantly worse Qmax when compared to TURP. TIND and PUL had the lowest Qmax change compared to TURP at 3 and 12 months, respectively (MD -9.94; 95%CI: -15.54, -4.33 and MD -9.60; 95%CI: -23.40, 4.20).

Ranking of MITs, TURP, and sham groups using the surface under the cumulative ranking (SUCRA) showed that Aquablation had the highest probability to improve Qmax (92.6%), followed closely by TURP (89.6%) at 3 months (Table-3). PAE, CWVTT, and PUL followed Aquablation and TURP, with TIND having the lowest probability of improving Qmax (23.1%). At 12 months, TURP had the highest probability to improve Qmax, followed by Aquablation, PAE and PUL, with probabilities of 79.0%, 69.9%, 31.7% and 19.4%, respectively. The certainty of evidence scored by GRADE is very low for TIND due to the high risk of bias and indirect comparison to TURP and low for all other MITs.

**Table 3:** SUCRA outcomes.

3 months	Qmax (%)	PVR (%)	IPSS (%)	QoL (%)
TURP	89.6	86.0	67.5	58.5
Aquablation	92.6	70.4	66.2	75.4
PUL	29.3	21.6	58.6	58.0
CWVTT	54.5	53.9	48.1	47.9
PAE	56.0	61.0	69.8	72.4
TIND	23.1	35.8	30.6	31.8
Sham	4.5	21.3	9.1	5.9
12 months	Qmax	PVR	IPSS	QoL
TURP	79.0	76.2	74.5	65.6
Aquablation	69.9	80.1	54.6	45.3
PUL	19.4	4.2	28.3	40.6
PAE	31.7	39.5	42.6	48.6

SUCRA = the surface under the cumulative ranking curve. The SUCRA is a calculation of the overall ranking in a single number. The higher the SUCRA the higher the chance that the treatment is in a high rank (equals a good outcome). The lower the SUCRA, the higher the chance that the treatment is in a low rank (equals a worse outcome).

#### Post Void Residual (PVR)

PVR improvement following Aquablation was most comparable to TURP at 3 and 12 months (MD 7.00; 95%CI: -32.14, 46.14 and MD -8.00; 95%CI: -53.17, 37.17) (Figures 3 C and D). PAE demonstrated a similar trend with a mean difference of 14.10; 95%CI: -7.75, 35.95 and -16.72; 95%CI: -6.90, 40.35 at 3 and 12 months, respectively. At 3 months, PVR following PUL was found to be significantly higher, indicating worse outcomes, compared to TURP (MD 44.45; 95%CI: 6.31, 82.59) (Figure-3C). However, at 12 months, there were no significant differences in PVR outcomes between MITs and TURP, although the mean difference between TURP and PUL was 60.10 (Figure-3D).

When ranking the treatments based on PVR change, TURP had the highest probability (86%) of improving PVR at 3 months (Table-3), followed by Aquablation with a probability of 70.4%. In contrast, PUL and the sham group were the least likely to improve PVR with probabilities of 21.6% and 21.3%, respectively. At 12 months, Aquablation had the highest probability (80.1%) to improve PVR, closely followed by TURP (76.2%). PUL was found to be the least likely to result in the best PVR improvement (4.2%). The certainty of evidence is very low for TIND and low for all other MITs.

#### IPSS

There were no significant differences in IPSS between MITs and TURP at 3 or 12 months (Figures 3E and F). However, at three months, the mean difference varied from -0.60 and -0.25 for Aquablation and PAE, respectively, to 3.96 and 7.42 for CWVTT and TIND, respectively. At 12 months, the mean difference for MITs varied between 1.10 and 3.40, compared to TURP (Figure-3F).

Ranking the treatments based on IPSS improvement did not show a clear preference (Table-3). The probabilities for best IPSS improvement by PAE, TURP and Aquablation were 69.8%, 67.5%, and 66.2%, respectively, at three months. Among the MITs, TIND (SUCRA 30.6%) was the least likely to improve IPSS. At 12 months, TURP had the highest probability to improve IPSS (74.5%). Of the MITs, Aquablation had the highest probability to improve IPSS (54.6%). The certainty of evidence is very low for TIND and low for all other MITs.

#### QoL

There were no significant differences in QoL scores between MITs and TURP at 3 and 12 months (Figures 3G and H). At three months, Aquablation and PAE showed a slightly lower mean difference than TURP (MD -0.50; 95%CI: -2.08, 1.08, MD -0.22; 95%CI: -1.09, 0.66). However, these differences were no longer present at 12 months (Figures 3G and H).

At three months, Aquablation and PAE had the highest probability of improving QoL, with 75.4% and 72.4%, respectively (Table-3). TURP, PUL, CWVTT and TIND followed. At 12 months, TURP had the highest probability of improving QoL (65.6%). The probability of PAE, Aquablation and PUL to result in the best QoL improvement was between 48.6% and 40.6%. The certainty of evidence is very low for TIND and low for all other MITs.

#### Sexual function

The incidence of erectile dysfunction *de novo* following PAE and TURP was 1% (n=2) and 3% (n=8), respectively, while no such cases were reported following any of the other MITs (Table-4). Retrograde ejaculation occurred in 14 (8%), 8 (7%), 4 (3%) and 67 (28%) patients following PAE, Aquablation, CWVTT, and TURP, respectively. Reduced ejaculate volume was reported in 3 (2%), 4 (3%) and 1 (0%) patient following PAE, CWVTT, and TURP, respectively.

**Table 4:** Adverse events, sexual function and treatment failure.

		Aquablation (n=116)	PUL (n=184)	CWVTT (n=134)	PAE (n=183)	TIND (n=128)	TURP (n=241)
Time period		90 days	NR	90 days	30-365 days	90 days	30-365 days
**Serious Adverse Events**		**7 (6%)**	**2 (1%)**	**3 (2%)**	**5 (3%)**	**5 (4%)**	**19 (8%)**
Bleeding		3 (3%)	1 (1%)	0 (0%)	0 (0%)	0 (0%)	5 (2%)
(Clot) retention		1 (1%)	1 (1%)	2 (1%)	0 (0%)	2 (2%)	1 (0%)
UTI/sepsis		0 (0%)	0 (0%)	0 (0%)	0 (0%)	3 (2%)	0 (0%)
Technical failure		0 (0%)	0 (0%)	0 (0%)	3 (2%)	0 (0%)	0 (0%)
Urethral stricture		3 (3%)	0 (0%)	0 (0%)	0 (0%)	0 (0%)	3 (1%)
Ischemia bladder wall		0 (0%)	0 (0%)	0 (0%)	1 (1%)	0 (0%)	0 (0%)
TUR syndrome		0 (0%)	0 (0%)	0 (0%)	0 (0%)	0 (0%)	1 (0%)
Other		0 (0%)	0 (0%)	1 (1%)	1 (1%)	0 (0%)	0 (0%)
**Adverse Events**		**83**	**286**	**138**	**305**	**83**	**317**
Post-embolization syndrome		0 (0%)	0 (0%)	0 (0%)	7 (4%)	0 (0%)	0 (0%)
Frequency		0 (0%)	0 (0%)	8 (6%)	15 (8%)	8 (6%)	0 (0%)
Dysuria		12 (10%)	82 (45%)	23 (17%)	18 (10%)	27 (21%)	63 (26%)
Haematuria		13 (11%)	53 (29%)	16 (12%)	10 (5%)	16 (16%)	67 (28%)
Haematospermia		0 (0%)	0 (0%)	10 (7%)	4 (2%)	0 (0%)	0 (0%)
Urgency		6 (5%)	10 (5%)	8 (6%)	0 (0%)	6 (5%)	4 (2%)
(Urge) incontinence		0 (0%)	6 (3%)	0 (0%)	0 (0%)	0 (0%)	16 (7%)
(Pelvic floor) pain		6 (5%)	25 (14%)	4 (3%)	21 (11%)	1 (1%)	34 (14%)
UTI		11 (9%)	7 (4%)	10 (7%)	12 (7%)	3 (2%)	33 (14%)
Epididymitis		0 (0%)	0 (0%)	4 (3%)	0 (0%)	0 (0%)	2 (1%)
Retention		11 (9%)	5 (3%)	5 (4%)	19 (10%)	8 (6%)	14 (6%)
Other		16 (13%)	15 (8%)	42 (31%)	180 (98%)	1 (1%)	29 (12%)
**Sexual function:**							
	Erectile dysfunction	0 (0%)	0 (0%)	0 (0%)	2 (1%)	0 (0%)	8 (3%)
	Retrograde ejaculation	8 (7%)	0 (0%)	4 (3%)	14 (8%)	0 (0%)	67 (28%)
	Reduced ejaculate volume	0 (0%)	0 (0%)	4 (3%)	3 (2%)	0 (0%)	1 (0%)
**Treatment failure at 1 year:**		3 (3%)	5 (3%)	0 (0%)	7 (4%)	3 (2%)	4 (2%)

CWVTT = Convective Water Vapor Thermal Therapy; NR = Not Reported; PAE = Prostatic Artery Embolization; PUL = Prostatic Urethral Lift; TURP = Transurethral Resection of the Prostate; UTI = Urinary Tract Infection

#### Adverse events

Serious adverse events (SAE) were reported following Aquablation, PUL, CWVTT, PAE, TIND, and TURP in 7 (6%), 2 (1%), 3 (2%), 5 (3%), 5 (4%), and 19 (8%) patients, respectively. An overview of (serious) adverse events following MITs and TURP is shown in Table-4. The most frequently occurring SAE following Aquablation and TURP were urethral stricture and bleeding. Technical failure was reported in only 3 (2%) PAE procedures due to iliac artery tortuosity or atherosclerotic changes (21). Urinary tract infection or sepsis incidence were more common following TIND (2%) (27).

Comparison of adverse events following MIT revealed that dysuria and hematuria occurred more frequently following PUL. The incidence of urine retention was two times higher in patients treated by Aquablation and PAE compared to PUL and CWVTT. In general, the number of adverse events following MITs was lower compared to TURP.

#### Retreatment rates

At one year follow-up, the retreatment rates were 3% (n=3), 3% (n=5), 0% (n=0), 4% (n=7),2% (n=3), and 2% (n=4) for Aquablation, PUL, CWVTT, PAE, TIND, and TURP, respectively (Table-4).

## DISCUSSION

This review provides an overview of several MITs that have become commercially available in the last 13 years and have been studied in RCTs. It presents treatment characteristics and safety outcomes, as well as functional outcomes compared to standard of care TURP, using an NMA at 3- and 12-months follow-up.

The MITs described in this review all have different approaches to improve voiding with subsequent varying side effects. The techniques vary in balance between the least invasive approach and best functional outcomes.

The results of this NMA showed that the functional outcomes following Aquablation were most comparable to TURP. This was seen in both objective outcomes by means of the Qmax and PVR as well as subjective outcomes by PROMs. PAE closely followed with slightly less improvement compared to TURP. PUL and TIND had the least voiding improvement compared to TURP. CWVTT outcomes fall between these groups for Qmax and PVR and are comparable with PUL and TIND for IPSS and QOL. SUCRA analysis confirmed the ranking of treatments described above.

Preservation of sexual function is one of the advantages of MITs, as mentioned in the included studies.

For some patient’s preservation of sexual function is of great importance and could be an important reason to choose an MIT over TURP. However, the variation in PROMs used by the included studies for sexual function evaluation, hampers outcome comparison by statistical analyses. Direct comparison of outcomes, although it should be interpreted with caution, seems to indicate a relation between the functional outcomes of a treatment and the chance of retrograde ejaculation. Consequently, TIND and PUL might be favored for sexual function preservation.

The improved safety profile is another often discussed advantage of MITs. The majority of MITs can be performed under local anesthesia or sedation, which eliminates potential general anesthesia side effects and makes them available for patients unfit for general anesthesia. Moreover, the majority of included MITs could be performed in an office-based setting or intervention room. This review confirms that MITs have a better safety profile than TURP, with TURP having the highest percentage of SAE’s, followed by Aquablation. The other MITs had lower SAE percentages, and AE rates were lower following MITs compared to TURP, except PUL which noted higher percentages for dysuria and hematuria, potentially due to the transurethral approach and insertion of material. Overall, the included MITs have a better safety profile than TURP.

The findings in this review are in line with findings of other recent MIT reviews, although it is the first to include PAE and compared to TURP. Tan-neru et al. included CWVTT, Aquablation and PUL in their systematic review and NMA and their findings were similar to those of this review (30). The Cochrane review by Franco et al. focused on PROM and adverse event outcomes, which indicated that IPSS for PAE and PUL were similar to TURP (31). However, the current review is unique as it covers all MITs including PAE and compared them to TURP for both objective and subjective outcomes. Therefore, this review provides a comprehensive overview and comparison of the novel MITs that have been investigated by RCT so far.

Our review has several limitations as a consequence of the scarcity of available literature. Since the included treatments are relatively new, only a limited number of RCT’s were available, subsequently leading to wide confidence intervals. Furthermore, the patient characteristics of the included patients showed some variation. Abt et al. included patients with better IPSS and QoL scores but worse Qmax and PVR, the impact of these contradictive baseline characteristics remains unclear. Furthermore, included studies had comparable inclusion and exclusion criteria, except for PUL that excluded median lobes. However, as baseline differences were limited, we assumed that in case there were effect modifiers that influenced the outcomes, these were distributed equally over the studies, and therefore transitivity is assumed. The network geometry showed that some comparisons were only based on a single study with limited patients, affecting the reliability of the network outcomes. Moreover, TURP outcome varied between PAE studies, suggesting heterogeneity between studies. In addition, the certainty of evidence is low to very low. For TIND the certainty of evidence is rated very low as the outcomes are only indirectly compared to TURP using the sham control group. Furthermore, the study had a high risk of bias. For the other studies, the outcomes were rated low due to the limited number of available studies with limited number of included patients. Therefore, the outcomes should be interpreted with caution. This review has not been able to provide high quality evidence to choose one MIT over another or over standard of care. As RCT’s are expensive additional RCT’s will be unfeasible. For these reasons, the overview of the currently available recent MITs for LUTS with their treatment effect and side effects provided by this review is the best achievable at the moment.

## CONCLUSIONS

Based on the findings in this review, it can be concluded that not all new minimally invasive treatments are equally effective in LUTS improvement. Of the reviewed MITs, Aquablation results in the best voiding improvement, outcomes are comparable to TURP. However, Aquablation is also the most invasive as it requires general or spinal anesthesia and hospitalization. If the desired technique can be performed under local anesthesia, PAE ranked as highest. These outcomes should be interpreted with caution due to the heterogeneity and low certainty of evidence of included studies.

## ABBREVIATIONS

BPH: Benign prostatic hyperplasia
CI: Confidence Interval
CWVTT: Convective Water Vapor Thermal Therapy
IIEF: International Index of Erectile Function
IPSS: International Prostate Symptom Score
LUTS: Lower Urinary Tract Symptoms
MIT: Minimally Invasive Treatment
NMA: Network Meta-Analysis
NR: Not Reported
PAE: Prostatic Artery Embolization
PUL: Prostatic Urethral Lift
PV: Prostate volume
PVR: Post-Void Residual
Qmax: Peak Urinary Flow
QoL: Quality of Life
RCT: Randomized Controlled Trial
SD: Standard deviation
SUCRA: Surface under the cumulative ranking
TIND: Temporary implantable nitinol device
TURP: Transurethral Resection of the Prostate
UTI: Urinary Tract Infection

## APPENDIX

### Supplementary 1 - Inclusion and exclusion criteria of patients per study.

**Table:**

Studies	Inclusion criteria	Exclusion criteria
Aquablation, Gilling et al. (4)	Men 45-80 years, Qmax ≤15, Prostate 30 - 80 cc by TRUS, IPSS ≥12	PVR >300 mL, Prostate/bladder cancer, Prior prostate surgery
PUL, Roehrborn et al. (2)	Men ≥ 50 years, Flow rate ≤12, VV ≥125 cc, AUASI ≥13, Prostate 30-80 cc, no prior surgical treatment for BPH, washouts of 2 weeks for a-blocker, 3 months for 5a-reductase inhibitor and 3 days for anticoagulants	Obstructive median lobe, PVR >250 mL, Active UTI, PSA >10 ng/mL (unless negative biopsy)
PUL, Sønksen et al. (11)	Male ≥ 50 years, Qmax ≤15 mL/s for 125 mL voided volume, IPSS >12, PVR <350 mL Prostate volume ≤ 60 cc, Incontinence Severity Index score ≤4	Median lobe, Active UTI, Urinary retention, Prior prostate surgery
CWVTT, McVary et al. (23)	Male ≥50 years, Qmax 5 - 15 mL/s for 125 mL voided volume, IPSS ≥13, Prostate volume 30 – 80cc, No prior invasive prostate intervention	PVR ≥250 mL, PSA ≥ 2.5 ng/mL, Active UTI
PAE, Insausti et al. (26)	Male >60 years, Qmax ≤10 mL/s or urinary retention, IPSS was ≥ 8, (QoL) related to LUTS was ≥ 3, TURP was indicated, BPH-related LUTS refractory to medical treatment for at least 6 months or the patient could not tolerate medical treatment	Advanced atherosclerosis, Tortuosity of the iliac arteries, GFR <30 mL/min, Neurogenic bladder, Prostate cancer
PAE, Abt et al. (25)	Male ≥ 40 years, Qmax <12 mL/s or urinary retention, IPSS was ≥ 8, Prostate 25 – 80 cc, QoL related to LUTS was ≥ 3, TURP indicated, refractory to medical treatment or not willing to undergo or continue medical treatment.	Advanced atherosclerosis, Tortuosity of the iliac arteries, GFR <60 mL/min, Neurogenic bladder, Prostate cancer
PAE, Gao et al. (21)	Qmax ≤ 15, (IPSS) >7, Prostate 20-100cc, Failed medical therapy with a washout period of 2 or more weeks.	Detrusor hyperactivity or hypocontractility at urodynamic study, Prostate cancer, Prior prostate surgery, PSA >4 ng/mL
PAE, Pisco et al. (28)	Male >45 yr old, Qmax <12 mL/s; IPSS of ≥ 20 and a QoL score of ≥ 3 PV ≥ 40 cc;	Prostatic arteries not feasible for PAE, Active UTI, Prior prostate surgery,
PAE, Carnevale et al. (24)	Male >45 yr old, IPSS >19, Prostate 30-90cc, bladder outlet obstruction (BOO) confirmed by urodynamic examination	Renal failure, suspected prostate cancer, neurogenic bladder disorder
TIND, Chughtai et al. (27)	Male ≥ 50 yr old, Qmax ≤ 12 mL/s, with 125mL voided volume, IPSS of ≥10, PV 25-75 cc;	PVR > 250mL, obstructive median lobe, PSA >10ng/mL or free PSA < 25% without a subsequent negative biopsy, previous prostate surgery.

CWVTT = Convective Water Vapor Thermal Therapy; IIEF = International Index of Erectile Function; IPSS = International Prostate Symptom Score; NR = Not Reported; PAE = Prostatic Artery Embolization; PV = prostate volume; PUL = Prostatic Urethral Lift; PVR = Post-Void Residual; Qmax = Peak Urinary Flow; QoL = Quality of Life; UTI = Urinary Tract Infection

### Supplementary 2 - Risk of Bias

**Table:** Qmax and post-void residual outcomes.

Studies	Intervention	Control	D1	D2	D3	D4	D5	O
Aquablation, Gilling et al. (4)	Aquablation	TURP	L	L	L	L	L	L
PUL, Roehrborn et al. (2)	PUL	Sham	L	L	S	L	S	S
PUL, Sønksen et al. (11)	PUL	TURP	L	S	S	L	L	S
CWVTT, McVary et al. (23)	CWVTT	Sham	L	L	L	L	L	L
PAE, Insausti et al. (26)	PAE	TURP	L	S	H	L	H	H
PAE, Abt et al. (25)	PAE	TURP	L	S	L	S	L	S
PAE, Gao et al. (21)	PAE	TURP	S	S	S	L	S	S
PAE, Pisco et al. (28)	PAE	Sham	L	L	L	S	S	S
PAE, Carnevale et al. (24)	PAE	TURP	S	S	L	S	S	S
TIND, Chughtai et al. (27)	TIND	Sham	L	H	H	L	S	H

**Table:** IPSS and QoL outcomes.

Studies	Intervention	Control	D1	D2	D3	D4	D5	O
Aquablation, Gilling et al. (4)	Aquablation	TURP	L	L	L	L	L	L
PUL, Roehrborn et al. (2)	PUL	Sham	L	L	L	L	S	S
PUL, Sønksen et al. (11)	PUL	TURP	L	S	S	L	L	S
CWVTT, McVary et al. (23)	CWVTT	Sham	L	L	L	L	L	L
PAE, Insausti et al. (26)	PAE	TURP	L	S	H	L	H	H
PAE, Abt et al. (25)	PAE	TURP	L	S	L	S	L	S
PAE, Gao et al. (21)	PAE	TURP	S	L	S	S	S	S
PAE, Pisco et al. (28)	PAE	Sham	L	L	L	S	S	S
PAE, Carnevale et al. (24)	PAE	TURP	S	S	S	S	S	S
TIND, Chughtai et al. (27)	TIND	Sham	L	S	H	S	S	H

**Table:** Adverse event outcomes

Studies	Intervention	Control	D1	D2	D3	D4	D5	O
Aquablation, Gilling et al. (4)	Aquablation	TURP	L	L	L	L	L	L
PUL, Roehrborn et al. (2)	PUL	Sham	L	L	L	L	S	S
PUL, Sønksen et al. (11)	PUL	TURP	L	S	S	L	L	S
